# Adiponectin, chemerin, cytokines, and dipeptidyl peptidase 4 are released from human adipose tissue in a depot-dependent manner: an in vitro system including human serum albumin

**DOI:** 10.1186/1472-6823-14-7

**Published:** 2014-01-22

**Authors:** Henrik Svensson, Birgitta Odén, Staffan Edén, Malin Lönn

**Affiliations:** 1 Department of Clinical Chemistry and Transfusion Medicine, Institute of Biomedicine, Sahlgrenska Academy, University of Gothenburg, S-405 30 Gothenburg, Sweden; 2 Department of Internal Medicine, Institute of Medicine, Sahlgrenska Academy, University of Gothenburg, S-405 30 Gothenburg, Sweden; 3 Department of Clinical Chemistry, Sahlgrenska University Hospital, Bruna stråket 16, S-413 45 Gothenburg, Sweden

**Keywords:** Adipokines, Cytokines, Subcutaneous adipose tissue, Visceral adipose tissue, Metabolic disease, Insulin resistance, Inflammation, Bovine serum albumin, Human serum albumin

## Abstract

**Background:**

Adipose tissue (AT) contributes to metabolic dysfunction through imbalanced production of adipokines, including cytokines. Visceral AT in particular is associated with metabolic disorders, indicating a specific secretory status. The relative significance of different human AT depots in adipokine release is not fully known. Further, previous in vitro systems usually included medium containing bovine serum albumin (BSA), which may induce cytokine release. Our aim was to compare release of a number of adipokines/cytokines – all implicated in insulin resistance – from human subcutaneous and visceral AT in a short-term incubation system minimizing cytokine induction and including repeated measurements during 24 h. A prerequisite was to evaluate a potential alternative to BSA in the incubation medium.

**Methods:**

Subcutaneous and/or visceral AT from 17 patients (age 20–68 years; BMI 22.6–56.7 kg/m^2^) undergoing elective surgery was incubated for 2, 4, 6, 8, and 24 h in medium with or without 1% BSA or human serum albumin (HSA). Medium concentrations of adiponectin, chemerin, nine cytokines, dipeptidyl peptidase 4 (DPP4), and omentin were analyzed by multiplex immunoassay or ELISA. Adipocyte size, AT macrophage density, and medium concentrations of endotoxin were determined.

**Results:**

Cytokine release was induced by BSA but not by HSA. In evaluation of the final incubation protocol including 1% HSA, and as expected, adiponectin release was higher from subcutaneous biopsies of nonobese than of obese subjects and inversely associated with adipocyte size; omentin was released almost exclusively from visceral AT. Exploratory incubations revealed more abundant release of chemerin, cytokines (except IL-6), and DPP4 from the visceral depot, while adiponectin release was higher from subcutaneous than visceral AT. Release was linear for a maximum of 2–6 h. Macrophage density was higher in visceral than subcutaneous AT. Levels of endotoxin in the medium were negligible.

**Conclusions:**

Adiponectin, chemerin, many cytokines, and DPP4 are released from human AT in a depot-dependent manner. These results highlight functional differences between visceral and subcutaneous AT, and a mechanistic link between regional fat accumulation and metabolic disorders. Supplementation of human AT incubation medium with HSA rather than BSA is recommended to minimize induction of cytokine release.

## Background

Adipose tissue is a major signaling organ that produces and releases many proteins with autocrine, paracrine, and endocrine activity, the so-called adipokines [1,2]. Dysregulated secretion of adipokines, caused by obesity and/or adipose tissue dysfunction, may contribute to the pathogenesis of metabolic disease, such as insulin resistance, type 2 diabetes, and cardiovascular disease [3]. Much attention has focused on adipokines with pro-inflammatory or anti-inflammatory activities since there is evidence of a connection between obesity, its co-morbidities, and signs of chronic low-grade inflammation [4,5].

Accumulation of visceral fat in particular is strongly associated with metabolic disorders [6,7], indicating that this depot may have a specific secretory status. Differences in cellular composition, adipocyte size, or other intrinsic factors could account for the regional specificity in adipokine release [8,9]. However, the relative significance of human subcutaneous and visceral adipose tissue in adipokine release has not been fully elucidated. Interestingly, gene expression studies revealed that expression of molecules involved in inflammation is higher in visceral adipose tissue [10-12]. However, differences in specific mRNA expression levels may not always be reflected in corresponding differences at the level of protein release. Short-term incubation of tissue pieces and subsequent analysis of the medium has been used to determine which proteins are actually released from adipose tissue depots and to what extent. Various systems for this purpose have been described that differ in supplementation of the medium, incubation time, proportion of tissue to medium, and other factors that may affect the outcome [13-17]. Thus, the challenge using an incubation strategy is to obtain results that reflect the in vivo situation [18].

Addition of bovine serum albumin (BSA) to the medium is often recommended in protocols for adipose tissue incubation, since albumin binds fatty acids that are mobilized and exert toxic effects when unbound [19]. In 2006 it was reported that BSA markedly induces production and release of cytokines from isolated human adipocytes [20]. This suggests that a BSA alternative not inducing cytokine release would be optimal, for example when comparing adipokine/cytokine release from specific adipose tissue depots [18]. Human serum albumin (HSA) has previously been used for supplementation of medium for both short-term and long-term incubation of human adipose tissue [16,21]. However, the influence of BSA versus HSA on cytokine release from human adipose tissue in vitro has not been assessed.

In this study, our aim was to compare the release of adipokines, including cytokines and other inflammatory/metabolic biomarkers, from human subcutaneous and visceral adipose tissue in a short-term incubation system minimizing induction of cytokine release, and including repeated measurements during 24 h. Further, a prerequisite was to first evaluate the influence of different albumin preparations regarding induction of cytokine release from human AT. Using our final incubation protocol, we investigated nine pro-inflammatory cytokines, the anti-inflammatory and intensively studied adiponectin, and chemerin and dipeptidyl peptidase 4 (DPP4), all with incompletely investigated, controversial or unexplored depot-dependent release.

## Methods

### Subjects

Adipose tissue biopsies were obtained from 17 patients (6 men and 11 women) undergoing elective abdominal surgery (abdominoplasty in 5, gall bladder removal in 2, hysterectomy in 1, and Roux-en-Y gastric bypass (RYGB) in 9). The mean age was 48.7 ± 13.1 yr (range 20–68 yr), and the mean BMI was 35.8 ± 10.8 kg/m^2^ (range 22.6–56.7 kg/m^2^). Subcutaneous abdominal adipose tissue biopsies were obtained from all subjects. Paired biopsies of subcutaneous and visceral (omentum) adipose tissue were collected from the 11 subjects (5 men and 6 women) undergoing gall bladder surgery or RYGB; these subjects had a mean age of 52.2 ± 15.1 yr (range 20–68 yr) and a mean BMI of 41.6 ± 8.9 kg/m^2^ (range 27.0–56.7 kg/m^2^). No subjects with malignant disease were included. All participants gave oral and written consent. The study was conducted in accordance with the Declaration of Helsinki and approved by the Regional Ethical Review Board, University of Gothenburg.

### Short-term incubation

Biopsies were initially handled as described in our protocol for long-term incubation [21,22]. In brief, pieces of adipose tissue (5–15 mg) were prepared under sterile conditions and incubated in plastic tubes (250 mg of tissue/10 mL of medium). In our final short-term incubation protocol, the tissue explants were incubated for 2, 4, 6, 8, and 24 h (one incubation tube for each incubation period) in Medium 199 (Invitrogen, Carlsbad, CA) supplemented with 30 mM NaHCO_3_, 0.15 μM adenosine, 1% HSA (Baxter Medical AB, Kista, Sweden), 0.1 mg/mL cephalotin (Eli Lilly, Indianapolis, IN), pH adjusted to 7.4. Medium was then removed and centrifuged for 5 min at 450 *g*. Aliquots of medium were stored at –80°C until analysis.

To evaluate the influence of albumin preparation, exploratory incubations for 2, 4, 6, 8, and 24 h were carried out in medium lacking albumin and in medium supplemented with 1% HSA, 1% BSA Fraction V, or 1% BSA Essentially Fatty Acid Free (Sigma-Aldrich, St Louis, MO).

The influence of 0.1% BSA was also studied. For practical reasons, only one tube of tissue was incubated with each medium, which was replaced after 4 h. After additional 20 h incubation, the second portion of incubation medium was removed.

All incubations were performed at 37°C in a 5% CO_2_ atmosphere in an incubator (Steri-cycle CO_2_ Incubator, Thermo Fischer Scientific, Marietta, OH); the medium was stirred gently with a rotator.

### Adipokine release

A multiplex immunoassay was used to measure the levels of nine cytokines in the incubation medium: granulocyte-macrophage colony-stimulating factor (GM-CSF), interferon-γ (IFN-γ), IL-1β, IL-2, IL-6, IL-8, IL-10, IL-12p70, and TNF-α (Human Pro-Inflammatory 9-Plex Ultra-Sensitive Kit, Meso Scale Discovery, Gaithersburg, MD). ELISA was used to measure adiponectin (Human adiponectin ELISA kit, Millipore, Billerica, MA), chemerin (Human chemerin ELISA kit, Millipore), omentin (Human omentin-1 ELISA, Millipore), IL-6 (Quantikine High Sensitivity Human IL-6 ELISA, R&D Systems, Minneapolis, MN), and DPP4 (Quantikine Human DPPIV/CD26 ELISA kit, R&D Systems). Adipokine concentrations were expressed per g adipose tissue.

### Adipocyte size

About 500 mg of each fresh biopsy was digested with collagenase [23], and the mean adipocyte diameter was determined by computerized image analysis [24] with Leica software (Leica QWin V3; Leica Microsystems, Wetzlar, Germany). The cell suspension was placed between a siliconized glass slide and a cover slip and transferred to the microscope (X5 objective, DM6000B, Leica Microsystems). Twelve random visual fields were photographed with a CCD-camera (DFC320, Leica Microsystems). Uniform microspheres, 98 μm in diameter (Dynal, Invitrogen Corporation, Oslo, Norway), served as reference. Mean adipocyte volume was calculated with the Goldrick formula [25].

### Immunohistochemistry

Paired subcutaneous and visceral adipose tissue specimens were fixed in phosphate-buffered formalin, dehydrated, embedded in paraffin and sectioned at 4 μm. After hydration and heat-induced antigen retrieval, a monoclonal antibody targeted at human CD68 was added to detect macrophages (Anti-Human CD68 clone PG-M1, DakoCytomation, Denmark). MACH 3 Mouse AP-Polymer and Warp Red was used as secondary and detection reagents (Biocare Medical Concord, CA). The sections were counter stained with hematoxylin and mounted. Slides were digitalized (Mirax Desk Digital Slide Scanner, Zeiss, Göttingen, Germany) and macrophages were counted and normalized for analyzed section area (Mirax Viewer, Zeiss). Origin of the tissue was unknown to the observer.

### Endotoxin in incubation media

The endotoxin concentrations in unconditioned medium with or without 1% of the different albumin preparations were analyzed at the Laboratory of Clinical Bacteriology, Sahlgrenska University Hospital, using a LAL assay with quantitative chromogenic end point (Limulus Amebocyte Lysate Endosafe Endochrome-K, Charles River Endosafe, Charleston, SC). The lower limit of detection was 0.5 pg/mL. All determinations were performed at least in duplicate.

### Statistical analysis

Values are expressed as mean ± SEM. To stabilize variances, values were logarithm-transformed. The influence of different albumin preparations (1%) on adipokine release over time was determined by three-way analysis of variance (medium, time, subject) followed by paired *t* test and Bonferroni correction. The influence of 0.1% BSA was evaluated by two-way analysis of variance (medium, subject) followed by Dunnett’s test. Differences in adipokine release from subcutaneous and visceral adipose tissue over time were assessed by three-way analysis of variance (depot, time, subject). To assess differences in adiponectin release between obese and nonobese subjects, we designed a split-plot experiment with subjects as main plots and times as subplots. Differences between groups were estimated from main plots, and differences between times were estimated from subplots [26]. Adipocyte size and macrophage density in subcutaneous and visceral adipose tissue was compared by paired *t* test. The relationship between adiponectin release and adipocyte size was investigated with Pearson correlation analysis. Simple regression was used to investigate the relationship between IL-6 concentrations in the medium determined by multiplex immunoassay and ELISA (non-transformed data). P < 0.05 was considered significant. All statistical analyses were conducted in SPSS (v18, SPSS, Chicago, IL).

## Results

### Effects of BSA and HSA on cytokine and adiponectin release from human adipose tissue

**Figure 1:**
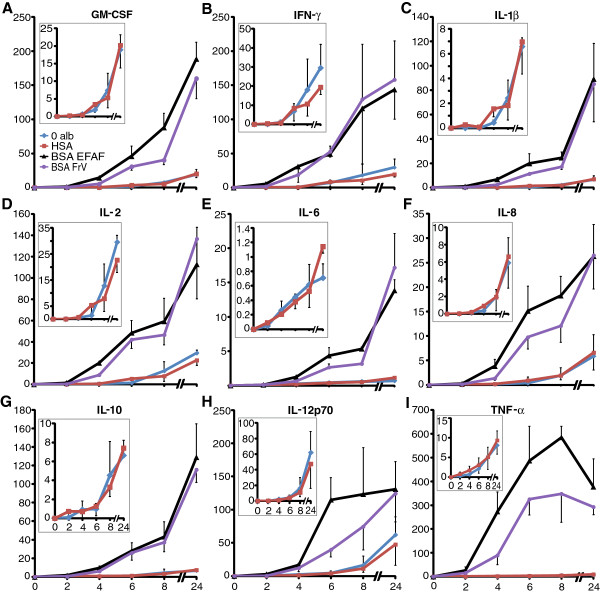
**Effects of BSA and HSA on cytokine release from human adipose tissue.** Adipose tissue was incubated for 2, 4, 6, 8, and 24 h in albumin-free medium (blue) and medium supplemented with 1% HSA (red), 1% BSA Fraction V (purple), or 1% BSA Essentially Fatty Acid Free (black). Concentrations of nine cytokines in the conditioned media were analyzed with a multiplex immunoassay; **(A)** granulocyte-macrophage colony-stimulating factor **(B)** interferon-γ **(C)** IL-1β **(D)** IL-2 **(E)** IL-6 **(F)** IL-8 **(G)** IL-10 **(H)** IL-12p70 **(I)** TNF-α. Insets show detailed plots of cytokine release in medium without albumin and in medium with HSA. Concentrations are ng/mL/g adipose tissue for IL-6 and IL-8 and pg/mL/g adipose tissue for the remaining cytokines. Values mean ± SEM (n = 3). Both media containing BSA markedly induced the release of all nine cytokines vs albumin-free medium or medium containing HSA over 24 h (p < 0.001). The two BSA preparations had similar effects, as did HSA and no albumin.

In three experiments, subcutaneous adipose tissue was incubated for 2, 4, 6, 8, and 24 h in albumin-free medium or medium supplemented with 1% HSA, 1% BSA Fraction V, or 1% BSA Essentially Fatty Acid Free. Both preparations of BSA markedly induced the release of all nine cytokines as analyzed by multiplex immunoassay (Figure 1). Release of all nine cytokines over 24 h was higher in the two media containing BSA than in albumin-free medium or medium containing HSA (p < 0.001). After 24 h, the mean cytokine concentration was 6- to 47-fold higher in media containing BSA than in medium containing HSA. TNF-α release at 24 h was 37-fold greater after incubation with BSA Fraction V and 47-fold greater after incubation with BSA Essentially Fatty Acid Free than after incubation with HSA. Cytokine concentrations did not differ between the two media containing BSA (Figure 1) or between albumin-free medium and medium containing HSA (Figure 1, insets). The adiponectin concentration in the medium was not influenced by albumin supplementation, as analyzed by specific ELISA (data not shown).

Medium supplemented with 0.1% BSA Fraction V and 0.1% BSA Essentially Fatty Acid Free also induced cytokine release. After 4 h, concentrations of all cytokines, except IL-2 and IL-6, were higher in both media with 0.1% BSA than in albumin-free medium (p < 0.05), as analyzed by multiplex immunoassay. After additional 20 h of incubation, concentrations of all nine cytokines were higher in both media with 0.1% BSA than in albumin-free medium (p < 0.05 IFN-γ, p < 0.001 all other cytokines) (data not shown).

### Endotoxin concentration in incubation media

The endotoxin concentration in a batch of unconditioned medium without albumin was <0.5 and 1.6 pg/mL (duplicate). In three batches of unconditioned medium supplemented with 1% HSA, the endotoxin concentration was 6.2, 9.1, and 3.8 pg/mL, respectively. The corresponding concentrations of endotoxin in medium containing 1% BSA Fraction V and 1% BSA Essentially Fatty Acid Free were 9200 and 11800 pg/mL.

### Adipocyte size in subcutaneous and visceral human adipose tissue

Adipocyte size in 11 paired biopsies of subcutaneous and visceral adipose tissue was analyzed with a computerized technique. The mean adipocyte volume was similar in subcutaneous and visceral adipose tissue (730 ± 160 vs 665 ± 217 pL, p = 0.153). The mean diameter of reference microspheres was 97.63 ± 0.25 μm (range 97.21–97.90 μm, n = 11). On average, 601 subcutaneous adipocytes (range 322–1245) and 515 visceral adipocytes (range 232–1164) were analyzed in each biopsy.

### Macrophage density in subcutaneous and visceral human adipose tissue

**Figure 2:**
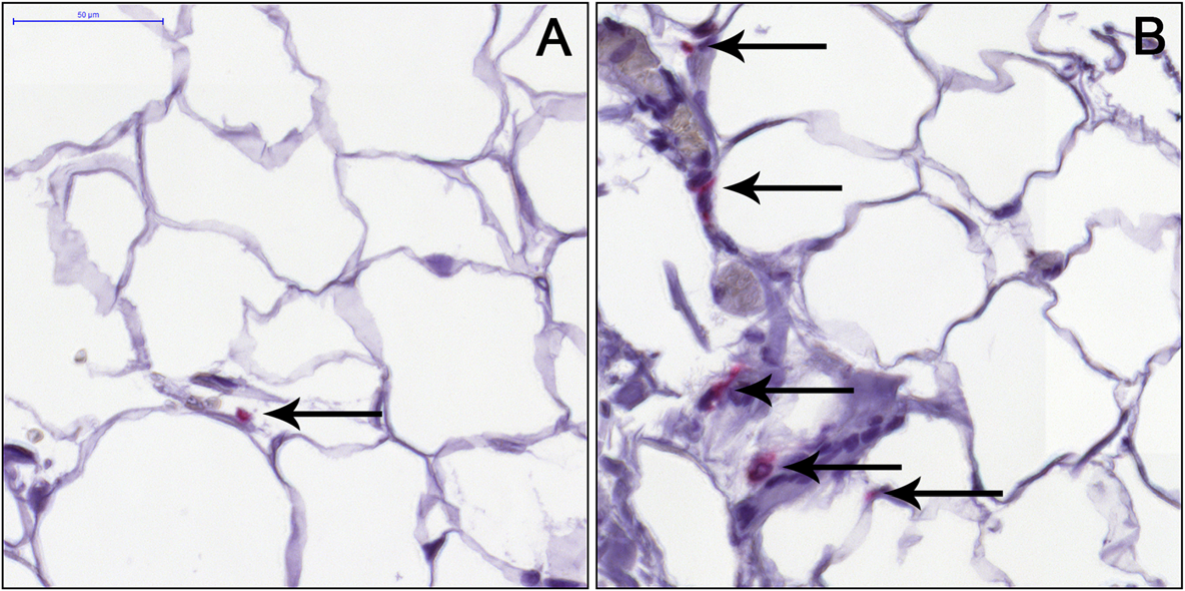
**CD68 immunoreactivity of human adipose tissue.** Positive signal appears red. Sections were counterstained with hematoxylin. Arrows indicate macrophages. Paired biopsies of **(A)** subcutaneous and **(B)** visceral adipose tissue (representative images).

The density of macrophages in 11 paired biopsies of subcutaneous and visceral adipose tissue was analyzed using immunohistochemistry. Macrophage density was higher in visceral than subcutaneous adipose tissue (1.74 ± 0.61 vs 0.39 ± 0.14 macrophages/mm^2^, p < 0.05) (Figure 2).

### Evaluation of the final short-term incubation protocol including HSA

**Figure 3:**
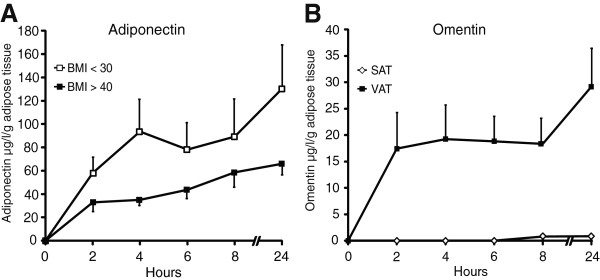
**Release of adiponectin and omentin from human adipose tissue.** Evaluation of the final incubation protocol including HSA. **(A)** Subcutaneous adipose tissue (SAT) from 10 subjects (5 obese and 5 nonobese) and **(B)** 11 paired biopsies of subcutaneous and visceral adipose tissue (VAT) were incubated for 2, 4, 6, 8, and 24 h in medium containing 1% HSA according to our final incubation protocol. The media were analyzed for **(A)** adiponectin and **(B)** omentin by ELISA. As expected, adiponectin was more abundantly released from adipose tissue of nonobese than obese subjects (p < 0.05) **(A)**, and omentin was almost exclusively released from the visceral depot (p < 0.001) **(B)**.

Subcutaneous adipose tissue from 10 subjects (five obese and five nonobese) was incubated for 2, 4, 6, 8, and 24 h in medium containing 1% HSA according to our final incubation protocol. Eleven paired biopsies of subcutaneous and visceral adipose tissue was also incubated accordingly. Adiponectin and omentin concentrations in the media were analyzed by ELISA. As expected, adiponectin was more abundantly released from adipose tissue of nonobese than of obese subjects (p < 0.05) (Figure 3A), and adiponectin release from subcutaneous adipose tissue was inversely associated with subcutaneous adipocyte size at 4 h (r = -0.85, p < 0.05, n = 7) and 24 h (r = -0.84, p < 0.05, n = 7). As expected, omentin was almost exclusively released from the visceral depot (p < 0.001) (Figure 3B).

### Release of cytokines from subcutaneous and visceral adipose tissue

Eleven paired biopsies of subcutaneous and visceral adipose tissue was incubated according to our final protocol. As shown by multiplex immunoassay, release of all nine cytokines but IL-6 were more abundant from visceral than subcutaneous adipose tissue over 24 h of incubation (GM-CSF p < 0.001, INF-γ p < 0.001, IL-1β p < 0.001, IL-2 p < 0.01, IL-6 p = 0.062, IL-8 p < 0.001, IL-10 p < 0.01, IL-12p70 p < 0.001, TNF-α p < 0.001) (data not shown).

### Release of adiponectin, chemerin, IL-6, and DPP4 from subcutaneous and visceral adipose tissue

**Figure 4:**
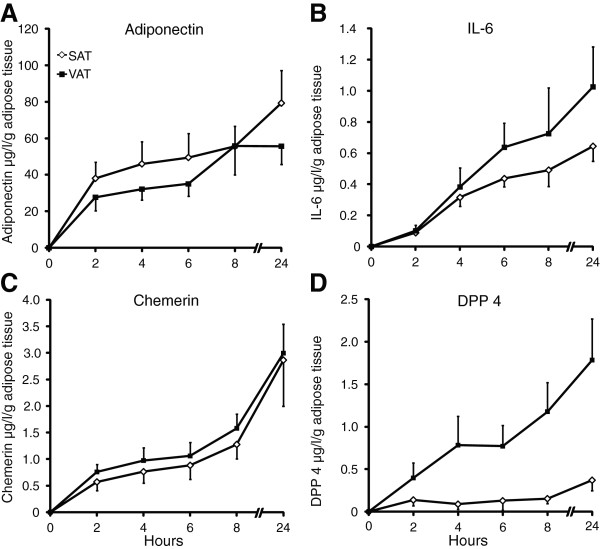
**Release of adiponectin, IL-6, chemerin, and DPP4 from subcutaneous adipose tissue (SAT) and visceral adipose tissue (VAT).** Concentrations of adiponectin, IL-6, chemerin and DPP4 in media from paired biopsies of subcutaneous and visceral adipose tissue incubated in medium containing 1% HSA according to our final protocol were determined by ELISA. Adiponectin was more abundantly released from subcutaneous than visceral adipose tissue (p < 0.01, n = 7) **(A)**. IL-6 release from the depots was similar (p = 0.315, n = 11) **(B)**, while chemerin **(C)** and DPP4 **(D)** were more abundantly released from visceral adipose tissue (p < 0.05 and p < 0.001, respectively, n = 11).

Paired biopsies of subcutaneous and visceral adipose tissue were incubated according to our final protocol, and concentrations of adiponectin, chemerin, IL-6, and DPP4 were determined by ELISA. Adiponectin was more abundantly released from subcutaneous than visceral adipose tissue (p < 0.01, n = 7) (Figure 4A). IL-6 release from the depots was similar (p = 0.315, n = 11) (Figure 4B), while chemerin and DPP4 were more abundantly released from visceral adipose tissue (p < 0.05 and p < 0.001, respectively, n = 11) (Figure 4C and 4D). Adipokine release was linear for a maximum of 2–6 h.

### Medium IL-6 concentrations determined by multiplex immunoassay and ELISA

The IL-6 concentration in 148 samples of medium were analyzed by ELISA and multiplex immunoassay. Regression analysis revealed a linear relationship without intercept (y = 0.714x, R = 0.987, p < 0.001), indicating almost complete agreement between the methods while the multiplex technique systematically underestimated the IL-6 concentration in the samples by about 30%.

## Discussion

This study shows that adiponectin, chemerin, eight pro-inflammatory cytokines, and DPP4, all inflammatory and/or metabolic biomarkers, are released from human adipose tissue in a depot-dependent manner. Chemerin, cytokines, and DPP4 were more abundantly released from visceral than subcutaneous adipose tissue, while adiponectin release was higher from the subcutaneous depot. Our findings also showed that cytokine release from human adipose tissue was markedly induced by BSA but not by HSA which was therefore used in our final protocol.

In particular, when comparing adipose tissue biopsies—from different groups of subjects, from different depots, or from an individual at different time points—with respect to adipokine/cytokine release, in vitro conditions must be standardized and selected with care [18]. In tests of our system, we found that HSA added to the medium, in contrast to BSA, did not induce cytokine release from human adipose tissue [20]. Using our final incubation protocol to monitor adipokine release over 24 h, we also obtained results consistent with previous well-established clinical findings [27-31]. First, adiponectin release was higher from abdominal biopsies of nonobese subjects than obese subjects [27,28,31]. Second, adiponectin release from subcutaneous adipose tissue was inversely associated with adipocyte size [29,30]. Further, omentin was released almost exclusively from visceral AT, consistent with reports that omentin mRNA is barely detectable in human subcutaneous adipose tissue or markedly lower in the subcutaneous than in the visceral depot [32,33]. Taken together, these findings suggest that our in vitro system is suitable for assessing the adipose tissue secretome and for investigating relative differences in adipokine/cytokine release from adipose tissue depots.

It may be noted that no differences in cytokine release was observed between medium containing HSA and medium without albumin. In a short-term incubation system like ours, with a high medium-to-adipose tissue ratio, supplementation of the medium with albumin may not be necessary.

As in a previous study of isolated adipocytes [20], cytokines were massively released when adipose tissue pieces were incubated in the presence of 0.1% or 1% BSA. Our results suggest that this effect of BSA may be mediated by a high concentration of endotoxin although immunomodulatory effects of albumin, not due to endotoxin contamination, also has been reported [34]. Depending on the cell/tissue type, endotoxin has a variety of effects mediated at the transcriptional and/or translational level. At low concentrations, the endotoxin lipopolysaccharide (LPS) reportedly increases the expression and release of cytokines from monocytes, monocyte-derived macrophages and in vitro differentiated adipocytes [35-37]. Levels of IL-8 mRNA in human mononuclear cells, cultured for 10 days for differentiation, were increased after a 4-h exposure to LPS at a concentration of 1 pg/mL [35]. However, relatively slow IL-8 protein secretion from the same cells was reported only after stimulation with 10 ng/mL LPS, making it difficult to draw conclusion about the LPS dose required for an effect on IL-8 release in this type of macrophage [35]. In our study, the endotoxin level was about 1 pg/mL in albumin-free medium and 3–10 pg/mL in incubation medium containing 1% HSA. Analysis of our conditioned media over 24 h revealed no differences in cytokine concentrations between media with or without HSA, despite the somewhat higher endotoxin level in medium containing HSA. This finding strongly suggests an absent or negligible effect of endotoxin, or other potential immunomodulatory feature of albumin, in our incubation system.

As mentioned above, measures of obesity are associated with reduced plasma levels of adiponectin, an adipokine predominantly produced by adipocytes [27,28,31,38]. Accumulation of visceral and truncal fat, in particular, may be linked to hypoadiponectinemia [28,39]. However, in vitro studies of adiponectin release from human subcutaneous and visceral adipose tissue have yielded inconsistent results. Adiponectin release from the depots was similar after incubation of tissue pieces for a couple of hours or for two days [40,41], while secretion of adiponectin was 28% higher from omental than subcutaneous adipocytes in primary culture, although the difference did not reach statistical significance [42]. In primary cultures of adipocytes from women with visceral obesity, adiponectin release was lower in omental than subcutaneous adipocytes [43]. Considerable methodological differences may underlie these discrepancies. Consistent with our findings, gene expression studies showed lower adiponectin mRNA levels in visceral than in subcutaneous adipose tissue in both lean and obese subjects [44,45].

To our knowledge, the possibility that release of chemerin is depot-dependent has not been investigated by incubation of human adipose tissue. Chemerin is a rather novel adipokine that regulates adipogenesis and may induce insulin resistance [46,47]. In obese subjects, elevated circulating levels correlate with BMI and measures of central adiposity, such as waist-hip ratio, waist circumference, and visceral adipose tissue mass [48]. Further, expression of chemerin mRNA in adipose tissue of patients with type 2 diabetes was more pronounced in the omentum than in the subcutaneous depot, suggesting a fat depot–dependent regulation of chemerin expression [49]. In the present study, the higher chemerin release from the visceral than the subcutaneous depot was marginal but still significant over 24 h.

DPP4, another recently discovered adipokine, may also impair insulin sensitivity [50]. Serum DPP4 is increased in obesity and reduced after weight loss and is a potential biomarker of metabolic syndrome [50]. In contrast, expression of DPP4 mRNA in subcutaneous adipose tissue was higher in lean women than in age-matched obese women [51]. In the present study, the first to investigate potential depot-dependent release of DPP4 from adipose tissue, visceral adipose tissue was by far the major site. This finding is consistent with a western blot analysis that showed higher DPP4 levels in visceral than in subcutaneous adipose tissue in obese subjects, and a similar trend also in lean subjects [50].

Accumulating evidence suggests that obesity—in particular central/visceral accumulation of fat—and its co-morbidities correlate with increases in circulating levels of inflammatory proteins such as C-reactive protein and IL-6 [4,5,52,53]. Plasma IL-6 concentrations were also reported to be higher in portal vein than in peripheral artery blood in obese subjects suggesting that visceral fat is an important source of IL-6 in obese people [54]. In vitro studies comparing release of proinflammatory cytokines such as IL-1β, IL-6, IL-8, and IL-18 from subcutaneous and visceral adipose tissue, usually in presence of BSA or fetal calf serum [13,15,53,55] but also in serum/albumin-free medium [13], generally show that the visceral depot is by far the dominant site [13,15,55,56], consistent with our findings. However, for TNF-α, similar release in the depots was previously reported [56,57]. Further, nonfat cells of the tissue are thought to be responsible for the major part of cytokine release [13,15,55], in line with our observation of higher macrophage density in the visceral depot. A larger number of observations would probably be required to demonstrate a significant association between macrophage density and cytokine release although we observed positive relationships (not shown). We found that release of IL-6, unlike the other cytokines we studied, was similar in the subcutaneous and visceral depots, although it tended to be higher in visceral adipose tissue. The reason for this is not clear but in light of previous clinical observations, and the fact that all other investigated cytokines in the present study were more abundantly released from the visceral depot, it is reasonable to assume that the non-significant difference between the depots regarding IL-6 release is due to the relatively small number of observations which limited our power.

Another possible limitation of this study is the rather heterogeneous group of patients included. However, analysis of paired biopsies from the obese subjects separately did not affect the results obtained. Further, the multiplex immunoassay is mainly intended for screening purposes. However, we observed an almost complete agreement between the multiplex immunoassay and the specific ELISA regarding analysis of IL-6 although the multiplex technique systematically underestimated the IL-6 concentration by about one third.

## Conclusions

Taken together, our findings show that human adipose tissue is characterized by depot-dependent release of many adipokines. Chemerin, GM-CSF, IFN-γ, IL-1β, IL-2, IL-8, IL-10, IL-12p70, TNF-α, and DPP4 were more abundantly released from visceral than subcutaneous adipose tissue, while adiponectin release was higher from the subcutaneous depot. These results highlight functional differences between visceral and subcutaneous adipose tissue, and a mechanistic link between regional fat accumulation and metabolic disorders. Our results also demonstrate that the system applied, including HSA instead of BSA, is suitable for further investigations of the human adipose tissue secretome and relative differences between depots. Further, for any in vitro system for human adipose tissue or adipocytes, potential albumin effects on tissue/cell function should be considered.

## Competing interests

The authors declare that they have no competing interests.

## Authors’ contributions

SE and ML designed the study. HS and BO collected patient material and performed the experiments. HS, SE and ML analyzed and interpreted the data. HS, BO, SE drafted the manuscript. ML revised the final version of the manuscript. All authors read and approved the final manuscript.

## Pre-publication history

The pre-publication history for this paper can be accessed here:

http://www.biomedcentral.com/1472-6823/14/7/prepub
